# The Comparative Effectiveness of Four Myopia-Control Spectacle Lenses: A Real-World AIPW Cohort Study

**DOI:** 10.3390/healthcare14182958

**Published:** 2026-09-10

**Authors:** Huan Xiao, Yichun Chai, Juan Wen, Qiulin Mi, Youruo Zhang, Junguo Duan

**Affiliations:** 1 School of Ophthalmology, Chengdu University of Traditional Chinese Medicine, Chengdu 610075, China; 2 Eye Health with Traditional Chinese Medicine Key Laboratory of Sichuan Province, Chengdu 610075, China

**Keywords:** myopia control, spectacle lenses, axial length, real-world evidence

## Abstract

**Purpose:** We aimed to compare the real-world effectiveness of four myopia-control spectacle lens interventions for childhood myopia control using a doubly robust causal inference framework. **Design:** This was a retrospective cohort study with multi-treatment augmented inverse probability weighting. **Participants:** A total of 2764 myopic children (right eyes) aged 3–18 years were included, screened from 13,071 electronic medical records at Ineye Hospital, Chengdu University of Traditional Chinese Medicine, between January 2023 and January 2026. **Methods:** Participants were allocated to four myopia-control spectacle lens groups and one single-vision spectacle lens group: Higher-Order Aberration Defocus *(n* = 912), Defocus Incorporated Multiple Segments (*n* = 800), Highly Aspherical Lenslets (*n* = 476), Diversified Segmental Defocus Optimization (*n* = 238), and single-vision lenses (*n* = 338). A doubly robust estimator combining multinomial propensity-score weighting with outcome regression was applied to adjust for confounding. **Outcome Measures:** The primary outcomes were one-year changes in axial length and spherical equivalent. The secondary outcomes were the rates of rapid progression. Treatment-effect heterogeneity was explored across prespecified baseline age, refractive error, and axial length subgroups. **Results:** After adjustment, all four myopia-control designs significantly outperformed single-vision correction (*p* < 0.05). The design based on Higher-Order Aberration Defocus demonstrated the greatest efficacy, with an axial length change of 0.107 mm and a spherical equivalent change of −0.146 D. Within this group, 25.5% of children exhibited rapid axial elongation (≥0.36 mm/year) and 8.3% exhibited rapid myopic progression (≥−0.50 D/year). The treatment effect was statistically significant in children with low myopia or an axial length no greater than 26 mm (*p* < 0.05), but not in those with high myopia or pathological axial elongation. **Conclusions:** All four myopia-control spectacle lens designs demonstrated significant advantages over single-vision correction in real-world clinical practice. Particular emphasis should be placed on the role of myopia-control spectacles during the early stages of myopia. As myopia progresses to high myopia or pathological axial elongation, the efficacy of myopia-control spectacle lenses as a standalone intervention diminishes, and combined approaches incorporating multiple myopia management strategies may be warranted.

## 1. Introduction

Myopia has become one of the most prevalent visual disorders of the modern era. Global projections indicate that by 2050, nearly half of the world’s population will be myopic, affecting approximately 5 billion people [1]. Among children and adolescents specifically, the prevalence is expected to reach 39.8% by 2050, with over 740 million young people affected [2]. The burden is disproportionately concentrated in East and Southeast Asia, where urban school-age children exhibit prevalence rates exceeding 80% in some cohorts [3]. Progressive high myopia is of particular clinical concern because irreversible axial elongation is strongly associated with a dramatically elevated lifetime risk of sight-threatening complications, including myopic maculopathy, retinal detachment, open-angle glaucoma, and cataract [4,5,6]. Therefore, effective strategies to slow myopia progression are of paramount importance.

Spectacle-based optical interventions have emerged as frontline myopia-control modalities owing to their excellent non-invasive safety profile, simplicity of use, and superior long-term adherence compared with contact lenses or pharmacotherapy [7]. Several optical architectures have been developed to impose myopic peripheral defocus signals on the retina, thereby modulating scleral remodeling through retinal growth-signaling pathways [8]. Among the most extensively evaluated are Defocus Incorporated Multiple Segments (DIMS) and Highly Aspherical Lenslets (HAL) spectacle lenses, each demonstrated to significantly slow axial elongation and refractive progression in randomized controlled trials (RCTs) [9,10]. More recently, Diversified Segmental Defocus Optimization (DSDO) lenses have been introduced, with initial RCT evidence supporting their efficacy in delaying myopia onset in premyopic children [11]. Emerging designs such as Higher-Order Aberration Defocus (HOAD) lenses have also shown promise in early studies [12]. However, direct head-to-head comparative evidence among these designs under routine clinical conditions remains limited.

Observational real-world evidence (RWE) studies are essential to complement RCT data, but they are inherently susceptible to confounding by indication, creating baseline imbalances that invalidate standard regression comparisons [13,14]. Augmented inverse probability weighting (AIPW) addresses this limitation through a doubly robust causal inference architecture that fuses propensity-score weighting with outcome regression modeling, yielding consistent average treatment effect (ATE) estimates when at least one of the two component models is correctly specified [15,16]. This framework is particularly suited to multi-treatment real-world comparisons because it preserves the entire study cohort and provides formal robustness guarantees against partial model misspecification.

The present study applies a multi-treatment AIPW framework to a large institutional cohort to compare the real-world effectiveness of four myopia-control spectacle lens interventions against single-vision correction in children with myopia.

## 2. Methods

### 2.1. Study Design and Ethics

**Figure 1:**
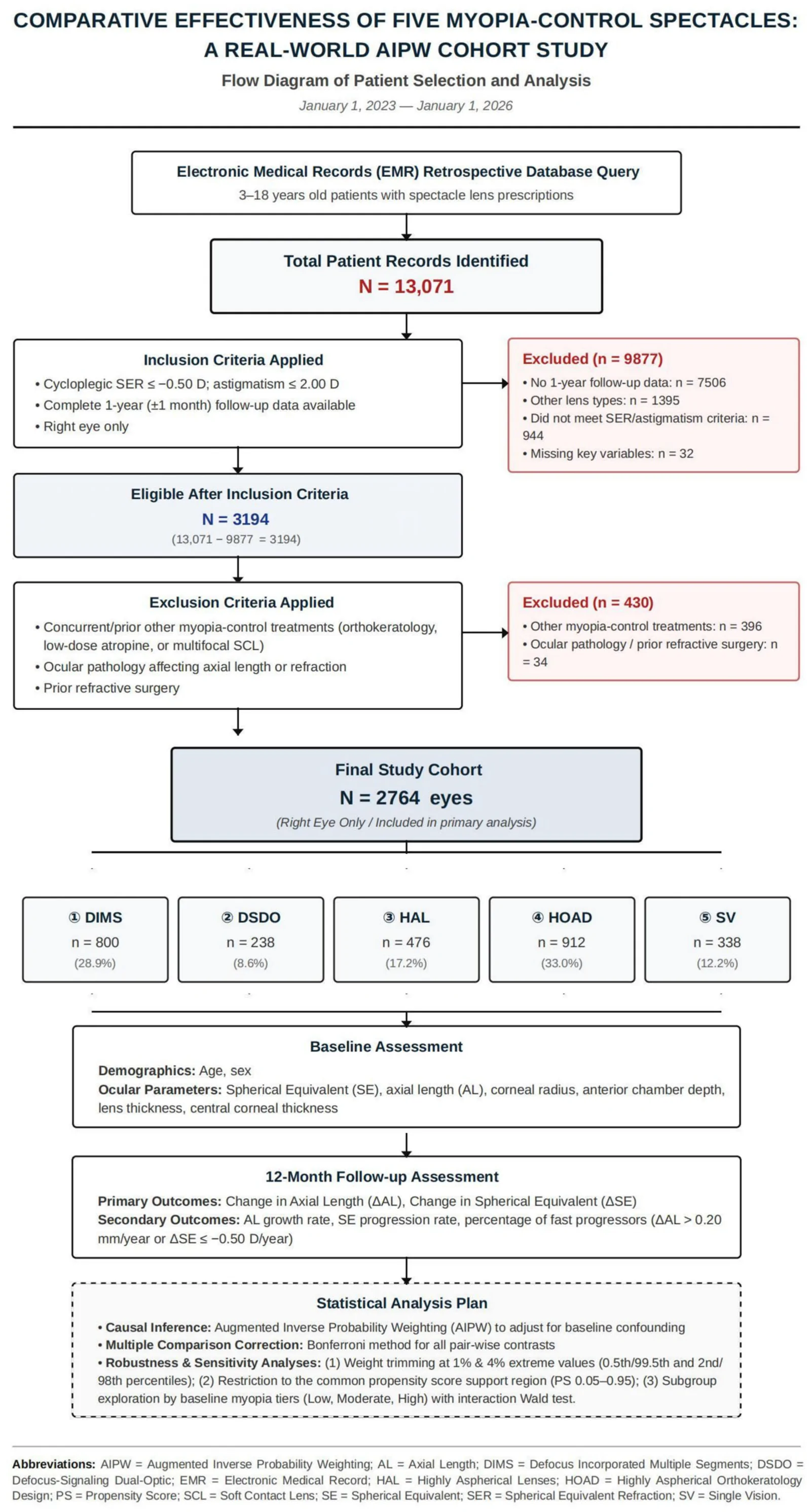
STROBE-compliant flowchart of participant screening and multi-treatment attrition. Sequential inclusion and exclusion criteria are presented with patient counts at each stage. The final cohort of 2764 right eyes was stratified into five intervention groups for primary analysis.

This retrospective, comparative cohort study was conducted at Ineye Hospital of Chengdu University of Traditional Chinese Medicine (Chengdu, China). The study was approved by the institutional review board (Approval No. 2025YH003) and adhered to the tenets of the Declaration of Helsinki. Individual informed consent was waived because of the retrospective, anonymized design. Participant screening and attrition are detailed in the STROBE-compliant flowchart (Figure 1). Reporting followed the STROBE guidelines for observational studies [17].

### 2.2. Participants

Electronic medical records for initial visits between 1 January 2023 and 1 January 2025 were screened, with 1-year follow-up assessments completed by 1 January 2026. From an initial base of 13,071 records, participants were excluded sequentially for absence of one-year follow-up data (*n* = 7506); non-study lens types (*n* = 1395); baseline cycloplegic spherical equivalent refraction (SE) > −0.50 D or astigmatism < −2.00 D (*n* = 944); missing baseline biometric variables (*n* = 32); concurrent or prior use of alternative myopia-control therapies (orthokeratology, low-dose atropine, or multifocal contact lenses) (*n* = 396); and prior refractive surgery including posterior scleral reinforcement (*n* = 34). All included participants had consistently worn their prescribed spectacle lens design for at least 1 year without lens replacement during the follow-up period. To eliminate inter-eye dependency, only the right eye of each participant was included. The final analytic cohort comprised 2764 children stratified by prescribed lens type: HOAD (*n* = 912), DIMS (*n* = 800), HAL (*n* = 476), single-vision (SV, *n* = 338), and DSDO (*n* = 238). In real-world clinical practice, spectacle lens allocation was not randomized but determined by a combined clinical and familial decision-making process based on baseline refractive error, axial elongation history, device availability, physician preference, and parental economic willingness. A detailed comparison of optical architectures, design mechanisms, and peripheral defocus profiles across all five spectacle lens groups is summarized in Supplementary Table S1.

### 2.3. Outcome Measures and Biometric Assessment

The primary endpoints were one-year changes in axial length (∆AL, mm) and cycloplegic refraction (∆SE, D). Secondary binary outcomes were the annual incidence of rapid axial progression (∆AL ≥ 0.2 mm/year) and rapid refractive progression (∆SE ≤ −0.75 D/year), calculated as the number of participants meeting the threshold divided by the total number of participants in each group, with thresholds representing clinically significant progression in school-age children [18,19]. The thresholds were selected as clinically relevant categorical indicators of relatively rapid progression and were used only for secondary binary outcomes; the primary analyses remained based on the continuous changes in axial length and spherical equivalent. The 0.20 mm/year AL threshold has been widely used as an indicator of clinically meaningful axial elongation in pediatric myopia studies, whereas the −0.75 D/year SE threshold was selected to identify relatively rapid refractive progression. Because progression rates vary substantially with age and baseline myopia severity, these binary thresholds were not used to define treatment efficacy across age groups; age-stratified analyses of the continuous ΔAL and ΔSE outcomes were additionally performed. To explore potential treatment-effect heterogeneity across clinically relevant patient subgroups, we conducted prespecified subgroup analyses stratified by age (≤6, 7–12, >12 years), baseline spherical equivalent (mild myopia: SE > −3.00 D; moderate myopia: −3.00 D to −6.00 D; high myopia: SE ≤ −6.00 D), and baseline axial length (normal/short: AL < 24.5 mm; elongated: 24.5–26.0 mm; pathologically elongated: AL > 26.0 mm). All biometric parameters—axial length (AL), mean corneal radius of curvature (R), anterior chamber depth (ACD), central corneal thickness (CCT), and crystalline lens thickness (LT)—were measured using high-precision optical biometry (IOLMaster 700; Carl Zeiss Meditec, Jena, Germany). All biometric parameters were measured at baseline and 1-year follow-up using the same IOLMaster 700 device (minimum 5 readings averaged, SNR > 2.0). Cycloplegic refraction was performed 30 min after 1% cyclopentolate instillation using auto-refraction (3 readings averaged), followed by subjective refinement with phoropter (RT-5100; NIDEK Co., Ltd., Gamagori, Japan) by certified optometrists.

### 2.4. Statistical Analysis

All analyses were conducted in R (v4.3; R Foundation for Statistical Computing, Vienna, Austria). The primary target estimand was the ATE in the overall eligible myopic pediatric population, with treatment defined according to the initially prescribed spectacle lens type. Propensity scores were estimated by multinomial logistic regression incorporating eight prespecified baseline covariates: age, sex, AL, SE, R, ACD, CCT, and LT. Multi-treatment AIPW estimators integrated these propensity scores with separate linear regression (for ∆AL and ∆SE) and logistic regression (for the rapid progression rate of AL and SE) outcome models [18,20]. This doubly robust architecture yields consistent ATEs when at least one component model is correctly specified.

Inverse-probability weights were trimmed at the 1st and 99th percentiles within each arm to mitigate the influence of extreme weights. Uncertainty was quantified by 500-replicate nonparametric bootstrap, with the entire AIPW pipeline re-estimated within each replicate. A fixed random seed (set.seed = 42) was used for reproducibility. A complete-case analysis was performed, with no missing-data imputation. No additional transformations or nonlinear terms were applied to the prespecified continuous covariates. Overall group differences were assessed by global Wald χ^2^ tests; pairwise comparisons were Bonferroni-corrected for multiple testing. For binary outcomes, AIPW-adjusted absolute risks were estimated, and NNT was derived as the reciprocal of the absolute risk difference between active treatment and single-vision controls. Uncertainty was quantified using 500 bootstrap replicates; if an absolute risk difference was non-significant (95% CI crossing zero), NNT was not interpreted as a single point estimate to avoid potential distortion. Covariate balance was assessed by standardized mean differences (SMDs), with SMD < 0.10 considered well balanced and SMD < 0.20 acceptable [20]. Positivity was assessed by visual inspection of the overlap in generalized propensity-score distributions across treatment groups.

Three prespecified sensitivity analyses were conducted to evaluate robustness: (1) weight trimming at the 0.5th and 99.5th percentiles; (2) trimming at the 2nd and 98th percentiles; and (3) restriction to the common propensity-score support region (0.05–0.95). The full results are reported in Supplementary Table S2.

## 3. Results

### 3.1. Baseline Characteristics and Confounding Diagnostics

**Figure 2:**
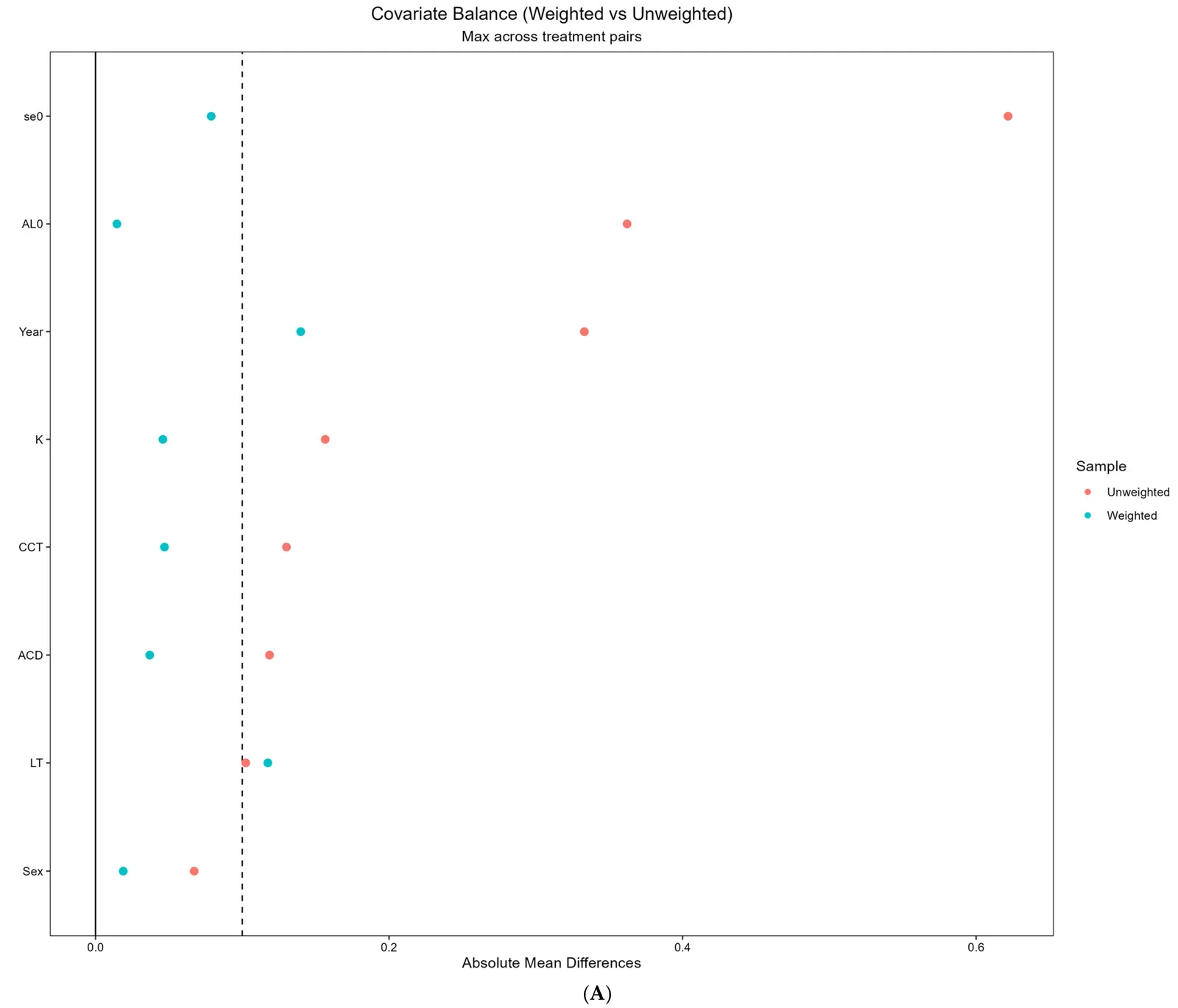

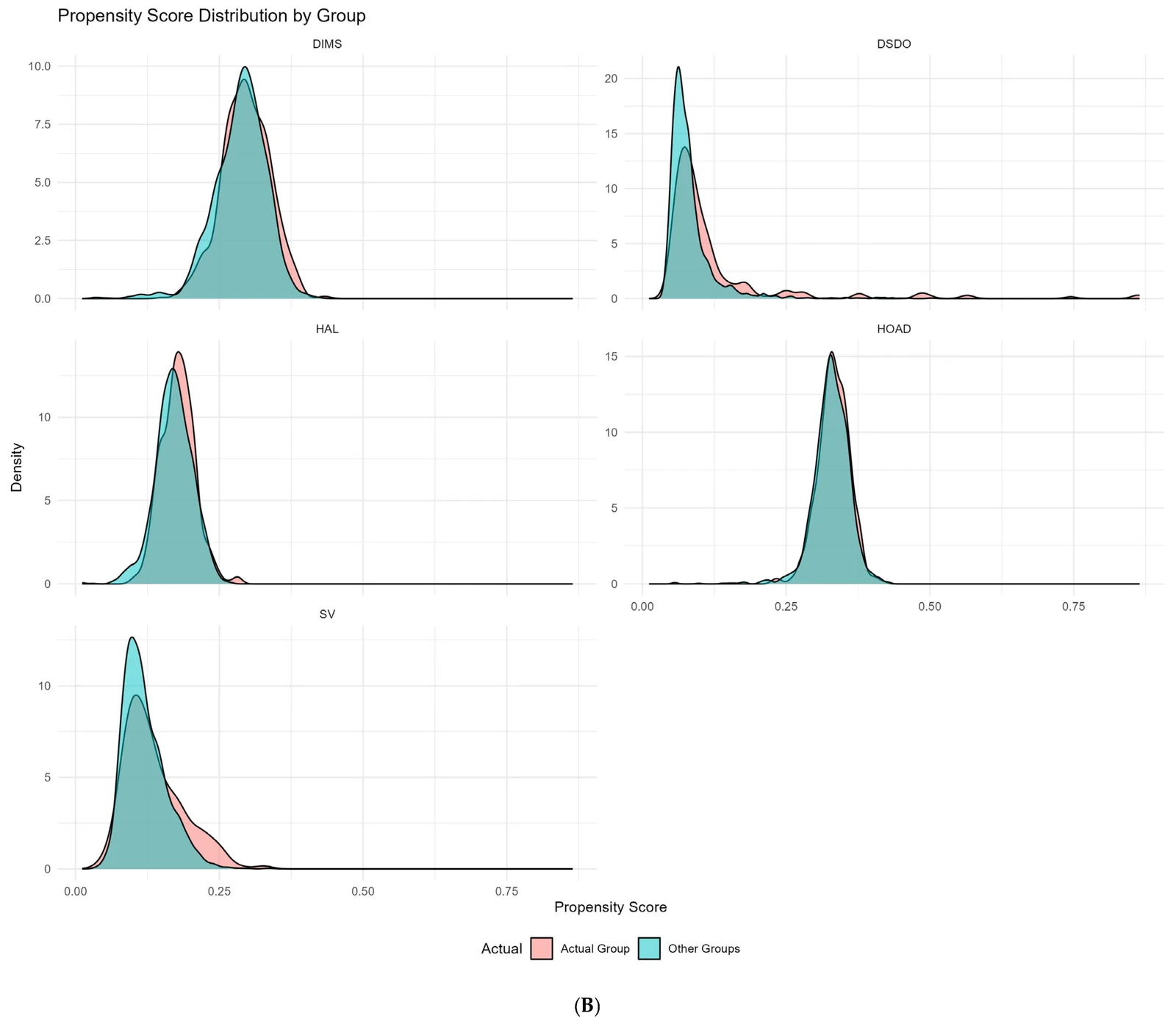
Covariate balance diagnostics after multi-treatment AIPW. (**A**) Love plot displaying maximum standardized mean differences (SMDs) across all treatment pairs before (salmon) and after (teal) weighting. The dashed vertical line represents SMD = 0.10. Note on interpretation: Points further to the right reflect greater baseline imbalances between treatment groups. Weighting (teal points) successfully shifts nearly all variables to the left of the dashed line (SMD < 0.10), indicating that baseline clinical differences between groups were effectively balanced, making the treatment groups directly comparable. (**B**) Propensity-score density distributions by treatment group, confirming substantial distributional overlap (positivity) across all five intervention arms.

Table 1 presents the baseline characteristics of the 2764 enrolled children. To ensure a fair clinical comparison despite baseline differences (e.g., initial age or axial length), statistical weighting (AIPW) was used to balance patient profiles across all groups. After balancing, clinical outcomes directly reflected lens efficacy rather than patient baseline differences. Before weighting, statistically significant imbalances were present across age, AL, R and SE (all *p* < 0.05), confirming strong confounding. After multi-treatment AIPW, covariate balance improved substantially: the majority of baseline parameters achieved SMD < 0.10, and residual imbalances in age and LT remained within the acceptable threshold of SMD < 0.20, as shown in the Love plot (Figure 2A). Detailed pairwise SMDs, effective sample sizes, and weight distributions are provided in Supplementary Tables S3 and S4. Propensity-score density distributions confirmed adequate positivity support across all five groups (Figure 2B).

### 3.2. Primary Continuous Outcomes (∆AL and ∆SE)

**Figure 3:**
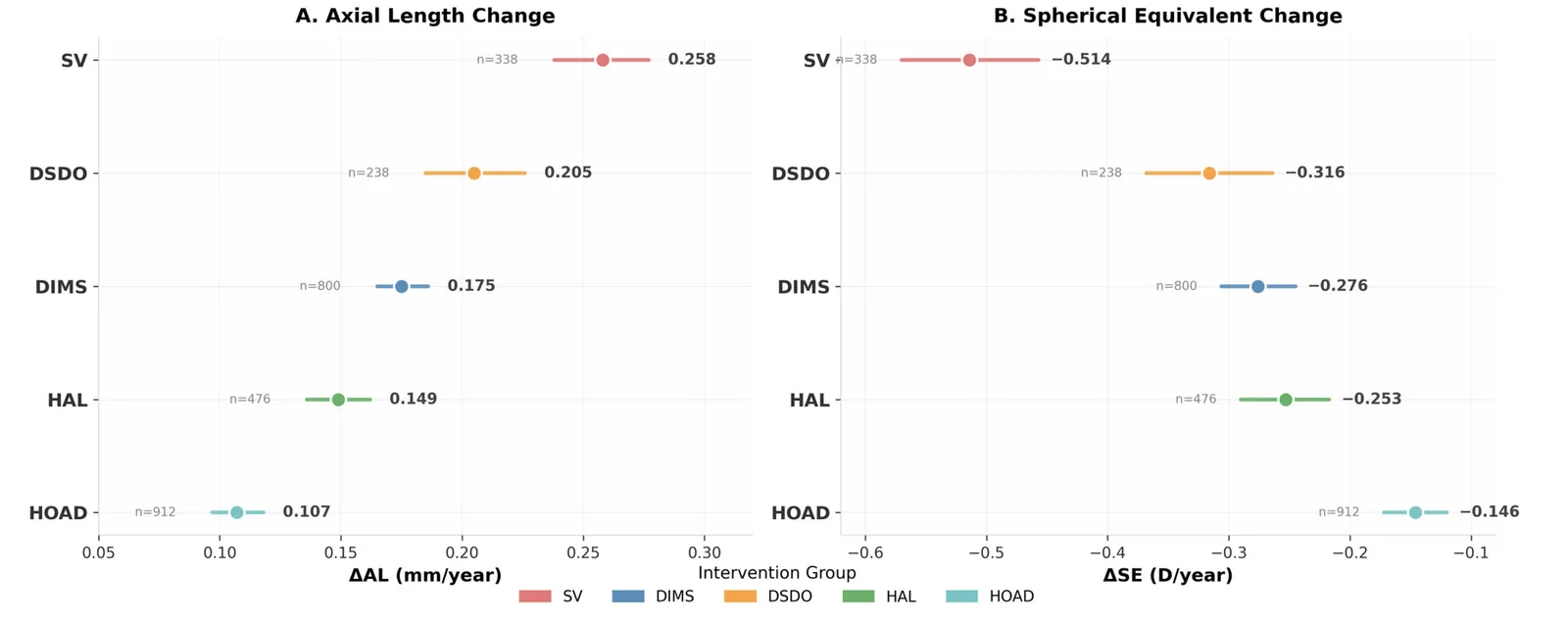
Marginal potential outcomes of ∆AL and ∆SE after AIPW adjustment. Points represent AIPW-estimated marginal means; error bars represent 95% bootstrap confidence intervals.

Clinically, the most relevant finding was that all four myopia-control lens designs were associated with smaller one-year axial elongation than SV correction, with the largest adjusted difference observed for HOAD. AIPW-adjusted marginal potential outcomes are summarized in Table 2 and illustrated in Figure 3. Compared with SV correction (∆AL: 0.258 mm/year; ∆SE: −0.514 D/year), all four myopia-control spectacle lenses significantly decelerated axial elongation and myopic shift (all *p* < 0.01). The ATEs for AL ranged from −0.052 mm/year (DSDO) to −0.150 mm/year (HOAD), and for SE from +0.198 D/year (DSDO) to +0.368 D/year (HOAD). HOAD demonstrated the most effective myopia control, with ∆AL of 0.107 mm, ∆SE of −0.146 D, and a 58.1% deceleration in annual axial elongation relative to SV.

### 3.3. Secondary Binary Outcomes: Rapid Progression Rates

**Figure 4:**
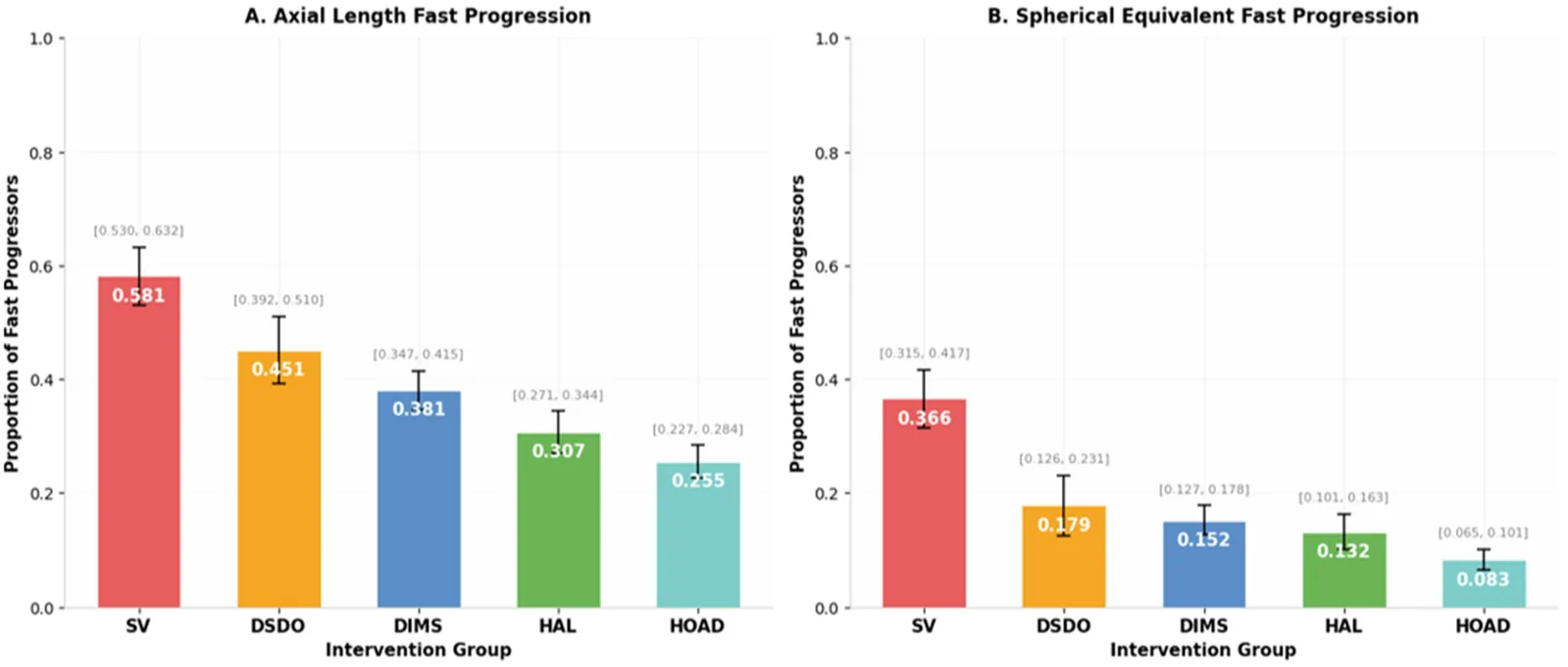
Rapid myopia progression rates across different spectacle lens groups.

The rates of rapid axial length progression (defined as ΔAL ≥ 0.2 mm/year) and rapid spherical equivalent progression (defined as ΔSE ≤ −0.75 D/year) are summarized in Table 3 and Figure 4. In the single-vision (SV) reference group, 58.1% of children exhibited rapid axial length progression and 36.6% showed rapid spherical equivalent progression over the 1-year follow-up period.

All four myopia-control spectacle lens designs were associated with significantly reduced risks of rapid progression compared with SV. For rapid axial length progression (ΔAL ≥ 0.2 mm/yr), all active lenses reduced absolute risks compared with SV (58.1%), yielding risk reductions of 13.0% for DSDO, 20.0% for DIMS, 27.4% for HAL, and 32.6% for HOAD. The ORs of rapid progression for SV relative to each intervention lens were 1.69 (DSDO), 2.25 (DIMS), 3.13 (HAL), and 4.04 (HOAD). These values indicate that the odds of rapid axial progression were higher in the SV group relative to each intervention. The NNTs to prevent one case of rapid axial length progression were 7.7 for DSDO, 5.0 for DIMS, 3.7 for HAL, and 3.1 for HOAD.

Similarly, for rapid spherical equivalent progression (ΔSE ≤ −0.75 D/yr), all active lenses reduced absolute risks compared with SV (36.6%), yielding risk reductions of 18.7% for DSDO, 21.4% for DIMS, 23.4% for HAL, and 28.3% for HOAD. The ORs of rapid progression for SV relative to each intervention lens were 2.65 (DSDO), 3.21 (DIMS), 3.79 (HAL), and 6.34 (HOAD). The corresponding NNTs were 5.3 for DSDO, 4.7 for DIMS, 4.3 for HAL, and 3.5 for HOAD.

HOAD demonstrated the most favorable outcomes, with the lowest rapid progression rates (25.5% for axial length and 8.3% for spherical equivalent), the lowest NNTs, and the highest odds ratios for SV relative to this lens.

### 3.4. Subgroup Analysis of Treatment Effects

**Figure 5:**
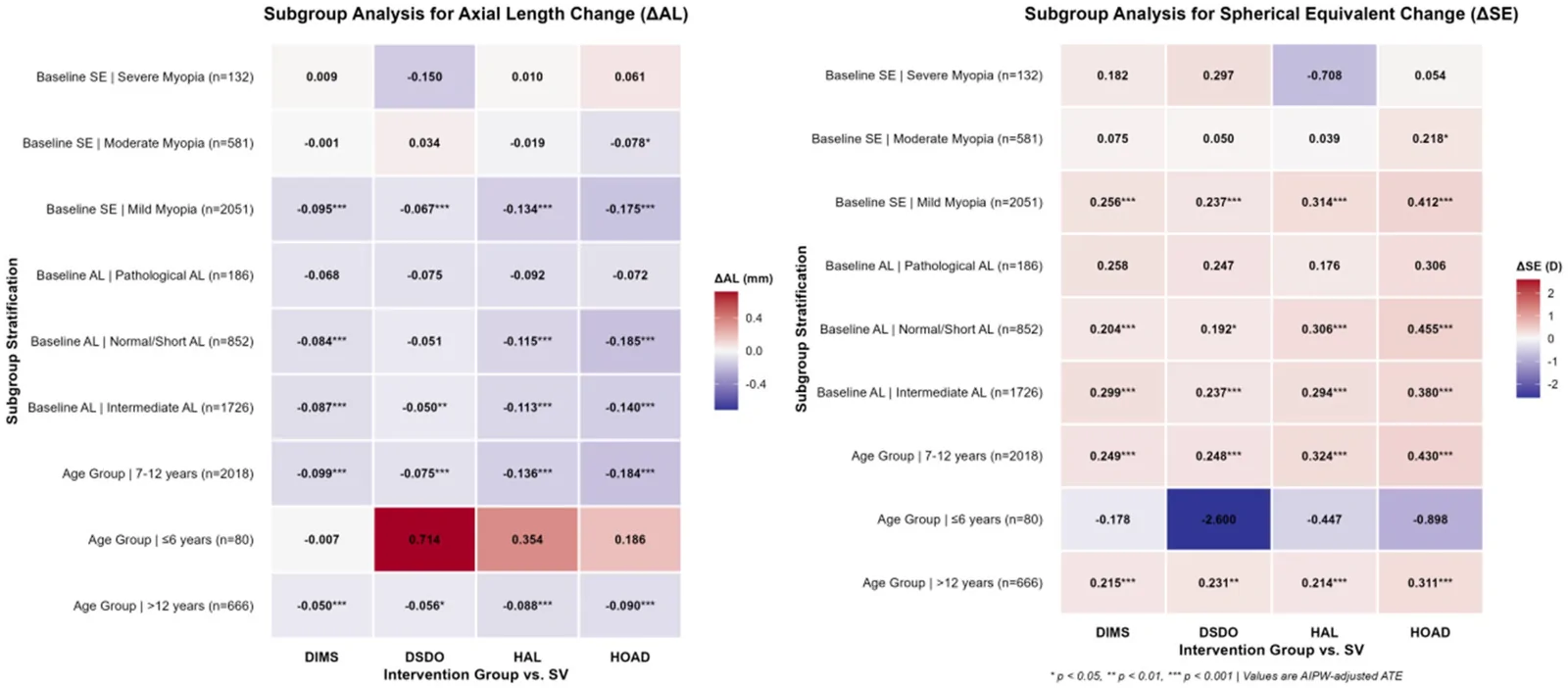
Subgroup analysis of axial length and spherical equivalent changes.

To investigate potential treatment-effect heterogeneity according to baseline characteristics, prespecified subgroup analyses were performed according to age, baseline SE, and baseline AL, and treatment-by-subgroup interactions were formally tested. Figure 5 presents the 1-year ATEs of the four myopia-control designs versus SV lenses on ΔAL and ΔSE. Formal tests of treatment-by-subgroup interactions showed significant interactions for age, baseline SE, and baseline AL for both ΔAL and ΔSE (all *p* < 0.05; Supplementary Table S6).

Age-stratified analysis. In children aged 6 years or younger (*n* = 80), the average treatment effects (ATEs) of the four myopia-control spectacle lenses versus single-vision (SV) correction did not reach statistical significance for either axial length or spherical equivalent (all *p* > 0.05), likely attributable to the limited sample size in this subgroup. In contrast, among children aged 7 to 12 years (*n* = 2018) and those older than 12 years (*n* = 666), all four lens designs demonstrated statistically significant ATEs compared with SV for both outcomes (all *p* ≤ 0.05).

Myopia-severity-stratified analysis. In the low-myopia subgroup (*n* = 2051), all four myopia-control lenses showed statistically significant ATEs versus SV for both axial length and spherical equivalent (all *p* ≤ 0.05). In the moderate-myopia subgroup (*n* = 581), only HOAD exhibited a statistically significant treatment effect. In smaller subgroups (such as children aged ≤6 years [*n* = 80], high myopia [*n* = 132], and pathological axial length [*n* = 186]), the sample sizes per active treatment arm were inherently constrained (e.g., *n* = 10 to 50; Supplementary Table S5). Consequently, the wide 95% CIs and non-significant ATEs observed in these strata primarily reflect low statistical precision and reduced power rather than definitive clinical ineffectiveness.

Axial-length-stratified analysis. In children with a normal or shorter axial length (*n* = 852) and those with a longer axial length (*n* = 1726), all myopia-control lenses except DSDO showed statistically significant ATEs versus SV for both axial length and spherical equivalent (all *p* ≤ 0.05); specifically, the ATE of DSDO for spherical equivalent did not reach statistical significance in these subgroups. In the pathologically elongated axial length subgroup (*n* = 186), no significant treatment effects were observed for any of the four lens designs.

### 3.5. Sensitivity Analyses

The results of the three prespecified sensitivity analyses are reported in Supplementary Table S2. Across all outcomes and analytical conditions, treatment-effect directions, efficacy rankings, and patterns of statistical significance were fully consistent with those observed in the primary analysis. No reversal of treatment effects was observed. Point estimates showed minimal variation across analytical strategies, indicating that the findings were robust to alternative propensity-score trimming specifications. Notably, these sensitivity analyses primarily assessed the influence of extreme weights and treatment-overlap assumptions and were not intended to eliminate residual confounding from unmeasured factors.

## 4. Discussion

Axial length has emerged as the definitive clinical endpoint for myopia management, as it is the primary structural determinant of myopia-related ocular morbidity. Epidemiological data demonstrate a steep dose–response relationship between increasing axial length and lifetime visual impairment risk: approximately 3-fold at 26–28 mm, 10-fold at 28–30 mm, and exceeding 90-fold at ≥30 mm [6]. As Brennan et al. have emphasized, even modest annual decelerations in axial elongation—when accumulated across the childhood progression window—represent clinically important reductions in the probability of reaching high myopia and its attendant morbidity [19]. Within this framework, comparative real-world evidence on the axial length efficacy of available spectacle-based interventions carries direct clinical relevance.

This study provides one of the first large-scale real-world comparative evaluations of five spectacle-based interventions for childhood myopia control using a causal inference framework. These findings represent comparative effectiveness estimates conditional on measured baseline confounders rather than definitive causal superiority. All four myopia-control spectacle lens designs demonstrated superior performance to SV correction in decelerating axial elongation and myopic refractive progression, with a consistent efficacy hierarchy observed across multiple outcome measures: HOAD showed the greatest effectiveness, followed by HAL, DIMS, and DSDO. This overall treatment ranking remained stable across sensitivity analyses, supporting the robustness of the findings despite the observational study design.

The real-world effectiveness of DIMS and HAL documented in this cohort validates and contextualizes established RCT evidence. Lam et al. demonstrated that DIMS lenses reduced myopic progression by 52% and axial elongation by 62% relative to SV controls over two years [9], with sustained efficacy confirmed through six years [21]. For HAL, Bao et al. demonstrated robust two-year axial growth reductions versus SV controls [10], subsequently confirmed over five years without evidence of rebound [22]. The present real-world data confirm that both designs deliver clinically meaningful myopia-slowing benefits in routine practice, supporting the external generalizability of these pivotal trials to heterogeneous clinical populations. The absolute magnitudes of treatment effects were modestly attenuated relative to the RCT findings—a well-recognized phenomenon attributed to variable wearing compliance, heterogeneous near-work and outdoor light exposure, and broader patient characteristics typical of clinical practice [13]. Nonetheless, the ATEs observed in this cohort are consistent with the pooled axial length treatment effect of approximately 0.15 mm reported in a recent meta-analysis of 21 randomized trials [23], suggesting that our estimates represent realistic clinical expectations.

Within this validated efficacy landscape, a clinically meaningful hierarchy emerged between the established designs. HAL consistently outperformed DIMS for axial elongation control and rapid-progression risk reduction—a finding concordant with the direct comparative study by Guo et al., which reported significantly slower axial elongation in children wearing HAL than those wearing DIMS over one year in an independent Chinese cohort [24]. The structural basis for this advantage likely resides in the spatial architecture of peripheral defocus: DIMS generates discrete focal points through spherical micro-lenslets separated by clear single-vision zones [9], whereas HAL employs contiguous, highly aspherical lenslets in concentric rings, producing a continuous volume of peripheral myopic defocus [10]. This continuous defocus volume may provide a spatially stable retinal signal. Nevertheless, the observational design precludes definitive mechanistic conclusions, and prospective trials incorporating peripheral wavefront aberrometry are required to confirm HOAD’s optical advantage. Although the between-group difference in annual axial elongation was modest, accumulated over the childhood progression window, this translates into a meaningfully lower lifetime risk of high myopia [19]. By contrast, pairwise differences between DIMS and DSDO were uniformly non-significant after Bonferroni correction in this cohort, suggesting broadly equivalent first-year outcomes. Notably, this finding differs from a recent 12-month prospective study reporting DSDO superiority over DIMS (*p* = 0.0202), highlighting the need for larger, longer-term comparative trials to resolve this discrepancy [25]. Given the limited DSDO sub-cohort size (*n* = 238) and single-year follow-up in the present study, longer-term multicenter studies are warranted to characterize its efficacy trajectory fully.

The most salient finding of this study is the consistently lower rate of axial elongation observed in the HOAD group across evaluated outcomes. HOAD achieved the smallest annual axial elongation (0.107 mm/year), the lowest refractive progression (−0.146 D/year), and the lowest probability of rapid progression (SV OR vs. HOAD 4.04), with all pairwise differences versus other active lens designs being statistically significant for axial elongation. To our knowledge, this is among the first real-world studies to characterize HOAD effectiveness in a multi-arm comparison using a rigorous causal inference approach. The mechanistic basis for HOAD’s superiority lies in its dual-mechanism optical framework combining peripheral myopic defocus with Higher-Order Aberration (HOA) modulation. While DIMS and HAL rely solely on peripheral myopic defocus—through discrete spherical micro-lenslets and contiguous aspherical lenslet rings, respectively [9,10]—HOAD additionally modulates peripheral spherical aberration and coma. If, as suggested by IMI experimental models, accommodation lag during sustained near work and oblique-gaze-induced aberrations contribute to peripheral hyperopic image degradation, then this dual strategy might address optical limitations that single-mechanism designs do not fully resolve [8]. These findings suggest that future spectacle lens development should consider multi-mechanism optical designs, as combining complementary optical strategies may potentiate myopia control efficacy. However, given the observational nature of our dataset, this optical mechanism remains a theoretical hypothesis that warrants validation through prospective wavefront aberrometry studies.

Our subgroup analyses revealed a loss in treatment efficacy with increasing myopia severity and axial elongation. This may be attributable to the more pronounced peripheral retinal stretching in high myopia, which could diminish the effectiveness of optical designs based on peripheral defocus principles. In a large-scale 1-year multicenter study, it was confirmed that higher baseline SER predicted poorer myopia control outcomes. The authors speculated that even the maximum +5.00 D myopic defocus embedded in the lens design may not provide a sufficient or effective defocus signal in subjects with increased baseline SER, as the relative peripheral refraction (RPR) profile in more myopic eyes tends to be more myopic, thereby partially neutralizing or overloading the imposed optical signal [26]. Beyond optical considerations, the biological plausibility of this diminishing returns phenomenon lies in the structural and biomechanical changes in the sclera in more advanced myopia. Myopia progression is fundamentally driven by scleral extracellular matrix (ECM) remodeling, characterized by reduced collagen synthesis, increased matrix metalloproteinase (MMP) activity, and decreased tissue inhibitor of metalloproteinase (TIMP) levels, leading to scleral thinning and biomechanical weakening [27]. In eyes with longer baseline AL and higher myopia, the sclera has already undergone substantial ECM degradation and biomechanical weakening. Consequently, the “inertia” of axial elongation is greater, and the same optical defocus signal may be less capable of reversing or halting the established pathological remodeling cascade. As noted in longitudinal orthokeratology studies, the first-year AL change has been shown to be a strong predictor of long-term progression, suggesting that the eye’s early response may reflect its sensitivity to biomechanical stimuli; once unfavorable scleral remodeling is established, the compensatory capacity of the choroid and retina to defocus signals diminishes [28]. Additionally, baseline RPR profiles differ across the myopia severity spectrum. Eyes with higher central myopia tend to exhibit more myopic RPR (i.e., greater relative peripheral myopia). When such eyes are fitted with defocus lenses providing a uniform +3.50 D peripheral myopic defocus, the net defocus signal at the peripheral retina may be excessive or poorly matched, thereby reducing treatment efficacy. This has been specifically demonstrated in children wearing DIMS lenses, where baseline myopic RPR was associated with significantly worse 2-year outcomes compared to baseline hyperopic RPR [29]. Therefore, particular emphasis should be placed on the early deployment of myopia-control spectacles during the initial stages of myopia development, when optical interventions are most effective. As myopia progresses to high myopia or pathological axial elongation, relying solely on spectacle-based optical interventions appears insufficient. In these advanced stages, combination approaches incorporating multiple myopia management modalities may be warranted to achieve meaningful therapeutic outcomes.

This study illustrates the value of AIPW for multi-treatment comparative effectiveness research in ophthalmology. Traditional propensity-score matching reduces analytical sample size and is difficult to extend beyond two treatment arms [11]. AIPW avoids these limitations by weighting the full cohort and simultaneously fitting outcome regression models, with formal doubly robust consistency properties [12,13]. While the stability of treatment rankings across sensitivity analyses supports the methodological consistency of the AIPW framework, these statistical adjustments cannot fully account for unmeasured behavioral variables, such as daily spectacle wear time, outdoor activity, or near-work intensity. Consequently, these findings should be interpreted as real-world clinical associations rather than definitive causal proof.

## 5. Limitations

Several limitations of this study merit explicit acknowledgment. First, marked disparities in sample size were observed across the intervention groups (ranging from *n* = 238 in the DSDO group to *n* = 912 in the HOAD group). This imbalance inherently reflects routine real-world clinical prescribing patterns, market availability, and parental or physician preferences during the study period. Although unequal sample sizes across treatment arms can potentially influence statistical power and variance estimation, our analytical framework explicitly mitigated this issue. Second, due to the retrospective nature of electronic medical record data, exact daily spectacle wear time (e.g., hours per day), adherence during specific activities (such as near work, visual display terminal use, or sports), and temporary non-wear periods caused by lost or damaged glasses were not systematically recorded. Variation in compliance and daily wearing habits is a well-recognized source of residual confounding in real-world evidence, which could have impacted treatment efficacy. Additionally, prior spectacle correction history before entering the cohort was not systematically recorded in the EMRs, which precludes assessment of potential carryover effects from previous lens designs. Third, although all spectacles were dispensed according to standard clinical protocols by certified optometric staff at a tertiary university eye hospital, procedural details regarding physical frame fitting—such as precise optical centration, pantoscopic tilt, back vertex distance, and differential vertical prism in anisometropic cases—were not standardized or tracked as study variables. Minor variations in these practical optical parameters could subtly influence the peripheral defocus profiles delivered to the retina. Fourth, despite doubly robust AIPW adjustment, the retrospective observational design cannot exclude residual confounding from unmeasured baseline environmental factors, such as daily outdoor light exposure, time spent on near work, and parental myopia status. Finally, the one-year follow-up period precludes the evaluation of long-term control efficacy, potential treatment deceleration, or rebound effects following treatment cessation.

## 6. Conclusions

In this large real-world cohort of 2764 myopic children, all four myopia-control spectacle lens designs significantly outperformed conventional single-vision correction in limiting one-year axial elongation and refractive progression. Among the evaluated designs, HOAD showed the strongest adjusted association with slowed axial elongation, followed by HAL, while DIMS and DSDO showed broadly comparable estimates after adjusting for measured confounders. The doubly robust AIPW framework successfully adjusted for pronounced channel-prescribing confounding and yielded estimates that were stable across all sensitivity analyses. These findings provide initial real-world comparative effectiveness estimates conditional on measured confounders to help generate hypotheses for pediatric myopia management. Prospective multicenter trials with longer follow-up and standardized compliance monitoring are warranted to confirm these observations. Given potential unmeasured behavioral confounders, these comparative estimates should be interpreted as hypothesis-generating and require confirmation in prospective multicenter studies.

## Figures and Tables

**Table 1:** Baseline characteristics of the study population by intervention group.

Variable	SV (*n* = 338)	HAL (*n* = 476)	DIMS (*n* = 800)	DSDO (*n* = 238)	HOAD (*n* = 912)	*p* Value
Male sex, *n* (%)	182 (53.8)	240 (50.4)	408 (51.0)	136 (57.1)	480 (52.6)	0.431
Age, yrs *	11 (9–14)	10 (9–12)	11 (9–12)	10 (9–12)	10 (9–12)	<0.001
AL, mm *	24.52 (23.84–25.10)	24.34 (23.89–24.97)	24.49 (23.88–25.16)	24.67 (24.05–25.33)	24.37 (23.79–25.08)	<0.001
R, mm *	7.80 (7.64–7.98)	7.85 (7.67–8.00)	7.84 (7.68–8.01)	7.80 (7.66–7.97)	7.80 (7.64–8.00)	0.035
ACD, mm	3.75 (3.56–3.92)	3.71 (3.58–3.85)	3.73 (3.58–3.91)	3.71 (3.56–3.87)	3.74 (3.59–3.87)	0.159
LT, mm	3.34 (3.24–3.44)	3.35 (3.25–3.44)	3.35 (3.25–3.46)	3.37 (3.25–3.46)	3.36 (3.26–3.46)	0.264
CCT, mm	0.54 (0.52–0.56)	0.54 (0.52–0.56)	0.54 (0.52–0.56)	0.54 (0.52–0.56)	0.54 (0.52–0.57)	0.319
SE, D *	−2.25 (−3.38, −1.38)	−2.00 (−3.00, −1.25)	−2.00 (−3.00, −1.25)	−2.75 (−4.13, −1.75)	−2.00 (−3.00, −1.25)	<0.001

Data presented as median (interquartile range). ACD = anterior chamber depth; CCT = central corneal thickness; LT = lens thickness; SE = spherical equivalent. * denotes statistically significant inter-group differences (*p* < 0.05; continuous variables were compared using Kruskal–Wallis tests and categorical variables using χ^2^ tests).

**Table 2:** AIPW-adjusted primary continuous outcomes and pairwise ATE vs. SV.

	Axial Length (mm/Year)		Spherical Equivalent (D/Year)	
Group	ΔAL (95% CI)	ATE vs. SV (95% CI)	ΔSE (95% CI)	ATE vs. SV (95% CI)
SV (reference)	0.258 (0.238, 0.277)	—	−0.514 (−0.570, −0.457)	—
DSDO	0.205 (0.185, 0.226)	−0.052 (−0.080, −0.024) **	−0.316 (−0.368, −0.264)	+0.198 (+0.121, +0.274) ***
DIMS	0.175 (0.165, 0.186)	−0.082 (−0.104, −0.060) ***	−0.276 (−0.306, −0.245)	+0.238 (+0.175, +0.301) ***
HAL	0.149 (0.136, 0.162)	−0.109 (−0.132, −0.085) ***	−0.253 (−0.290, −0.217)	+0.260 (+0.193, +0.327) ***
HOAD	0.107 (0.097, 0.118)	−0.150 (−0.172, −0.128) ***	−0.146 (−0.172, −0.120)	+0.368 (+0.306, +0.430) ***

Values are AIPW-estimated marginal means with 95% bootstrap confidence intervals (500 replicates). ATE = average treatment effect (active group minus SV); CI = confidence interval; AL = axial length; SE = spherical equivalent. ** *p* < 0.01, *** *p* < 0.001 after Bonferroni correction. A positive ∆AL reflects axial elongation, and a negative ∆SE reflects myopic progression. ATE = active lens group.

**Table 3:** Rapid progression rates, odds ratios (SV vs. intervention), and number needed to treat vs. SV.

	Rapid Progression—ΔAL ≥ 0.2 mm/yr			Rapid Progression—ΔSE ≤ −0.75 D/yr		
Group	Rate	SV OR vs. Intervention (95% CI)	NNTs	Rate	SV OR vs. Intervention (95% CI)	NNTs
SV (reference)	58.1%	—	—	36.6%	—	—
DSDO	45.1%	1.69 (1.23–2.31) **	7.7	17.9%	2.65 (1.73–4.04) ***	5.3
DIMS	38.1%	2.25 (1.74–2.92) ***	5.0	15.2%	3.21 (2.41–4.27) ***	4.7
HAL	30.7%	3.13 (2.35–4.16) ***	3.7	13.2%	3.79 (2.67–5.38) ***	4.3
HOAD	25.5%	4.04 (3.14–5.21) ***	3.1	8.3%	6.34 (4.62–8.72) ***	3.5

OR = odds ratio; CI = 95% bootstrap confidence interval; NNT = number needed to treat (to prevent one additional case of rapid progression vs. single-vision control). ** *p* < 0.01. *** *p* < 0.001 after Bonferroni correction.

## Data Availability

The data presented in this study are available on request from the corresponding author. The data underlying this article cannot be shared publicly due to privacy and ethical restrictions.

## Supplementary Materials

The following supporting information can be downloaded at: https://www.mdpi.com/article/10.3390/healthcare14182958/s1. Supplementary Table S1: Comparison of optical architecture among five spectacle lens groups. Supplementary Table S2: Sensitivity analyses of pairwise comparisons across outcomes. Supplementary Table S3: AIPW Diagnostic Summary: Covariate Balance, Weight Distribution, Effective Sample Size, and Positivity Assessment. Supplementary Table S4: Pairwise Covariate-Specific Standardized Mean Differences (SMD), Pre- and Post-AIPW Weighting. Supplementary Table S5: Subgroup analysis of the 1-year ATEs of the four myopia-control designs versus SV lenses on ΔAL and ΔSE. Supplementary Table S6: Tests for interaction between treatment assignment and subgroup variables on 12-month axial length and spherical equivalent changes.

## References

[1] Holden B.A., Fricke T.R., Wilson D.A., Jong M., Naidoo K.S., Sankaridurg P., Wong T.Y., Naduvilath T.J., Resnikoff S. (2016). Global prevalence of myopia and high myopia and temporal trends from 2000 through 2050. Ophthalmology.

[2] Liang J., Pu Y., Chen J., Liu M., Ouyang B., Jin Z., Ge W., Wu Z., Yang X., Qin C. (2025). Global prevalence, trend and projection of myopia in children and adolescents from 1990 to 2050: A systematic review and meta-analysis. Br. J. Ophthalmol..

[3] Pan Z., Xian H., Li F., Wang Z., Li Z., Huang Y., Liu W., Li Y., Li F., Wang J. (2025). Myopia and high myopia trends in Chinese children and adolescents over 25 years: A nationwide study with projections to 2050. Lancet Reg. Health West Pac..

[4] Haarman A.E., Enthoven C.A., Tideman J.W.L., Tedja M.S., Verhoeven V.J., Klaver C.C. (2020). The complications of myopia: A review and meta-analysis. Invest Ophthalmol. Vis. Sci..

[5] Bullimore M.A., Ritchey E.R., Shah S., Leveziel N., Bourne R.R., Flitcroft D.I. (2021). The risks and benefits of myopia control. Ophthalmology.

[6] Tideman J.W.L., Snabel M.C., Tedja M.S., Van Rijn G.A., Wong K.T., Kuijpers R.W., Vingerling J.R., Hofman A., Buitendijk G.H., Keunen J.E. (2016). Association of Axial Length with Risk of Uncorrectable Visual Impairment for Europeans with Myopia. JAMA Ophthalmol..

[7] Wolffsohn J.S., Kollbaum P.S., Berntsen D.A., Atchison D.A., Benavente A., Bradley A., Buckhurst H., Collins M., Fujikado T., Hiraoka T. (2019). IMI—Clinical myopia control trials and instrumentation report. Investig. Ophthalmol. Vis. Sci..

[8] Troilo D., Smith E.L., Nickla D.L., Ashby R., Tkatchenko A.V., Ostrin L.A., Gawne T.J., Pardue M.T., Summers J.A., Kee C.S. (2019). IMI—Report on experimental models of emmetropization and myopia. Investig. Ophthalmol. Vis. Sci..

[9] Lam C.S.Y., Tang W.C., Tse D.Y.Y., Lee R.P.K., Chun R.K.M., Hasegawa K., Qi H., Hatanaka T., To C.H. (2020). Defocus Incorporated Multiple Segments (DIMS) spectacle lenses slow myopia progression: A 2-year randomised clinical trial. Br. J. Ophthalmol..

[10] Bao J., Huang Y., Li X., Yang A., Zhou F., Wu J., Wang C., Li Y., Lim E.W., Spiegel D.P. (2022). Spectacle lenses with aspherical lenslets for myopia control vs single-vision spectacle lenses: A randomized clinical trial. JAMA Ophthalmol..

[11] Lu Y., Yang X., Zhou J., Chen S., Li X., Deng Y., Zhang Y., Li Y., Wang K. (2025). Diversified segmental defocus optimization lenses with and without atropine for myopia prevention: A randomized clinical trial. JAMA Ophthalmol..

[12] Guo X., Li M., Zhang K., Lv Y., Lu W., Chen X., Li L. High order aberration diffusion spectacle lenses for myopia control versus defocus incorporated multiple segment spectacle lenses: A prospective randomised non-inferiority trial. Proceedings of the ARVO Annual Meeting.

[13] Sherman R.E., Anderson S.A., Dal Pan G.J., Gray G.W., Gross T., Hunter N.L., LaVange L., Marinac-Dabic D., Marks P.W., Robb M.A. (2016). Real-world evidence—What is it and what can it tell us?. N. Engl. J. Med..

[14] Stuart E.A. (2010). Matching methods for causal inference: A review and a look forward. Stat. Sci..

[15] Robins J.M., Rotnitzky A., Zhao L.P. (1994). Estimation of regression coefficients when some regressors are not always observed. J. Am. Stat. Assoc..

[16] Zhong Y., Kennedy E.H., Bodnar L.M., Naimi A.I. (2021). AIPW: An R package for augmented inverse probability–weighted estimation of average causal effects. Am. J. Epidemiol..

[17] Von Elm E., Altman D.G., Egger M., Pocock S.J., Gøtzsche P.C., Vandenbroucke J.P. (2007). The Strengthening the Reporting of Observational Studies in Epidemiology (STROBE) statement: Guidelines for reporting observational studies. Lancet.

[18] Tideman J.W.L., Polling J.R., Vingerling J.R., Jaddoe V.W., Williams C., Guggenheim J.A., Klaver C.C. (2018). Axial length growth and the risk of developing myopia in European children. Acta Ophthalmol..

[19] Brennan N.A., Toubouti Y.M., Cheng X., Bullimore M.A. (2021). Efficacy in myopia control. Prog. Retin Eye Res..

[20] Austin P.C. (2009). Using the standardized difference to compare the prevalence of a binary variable between two groups in observational research. Commun. Stat. Simul. Comput..

[21] Lam C.S.Y., Tang W.C., Zhang H.Y., Lee P.H., Tse D.Y.Y., Qi H., Vlasak N., To C.H. (2023). Long-term myopia control effect and safety in children wearing DIMS spectacle lenses for 6 years. Sci. Rep..

[22] Li X., Huang Y., Liu C., Chang X., Cui Z., Yang Q., Drobe B., Bullimore M.A., Chen H., Bao J. (2025). Myopia control efficacy of spectacle lenses with highly aspherical lenslets: Results of a 5-year follow-up study. Eye Vis..

[23] D’Andrea L., Rinaldi M., Piscopo R., Iorio F., La Padula S., Maqsood S., Elalfy M., Melenzane A., Confalonieri F., Ozkan O. (2026). Efficacy of spectacle lenses for myopia control: A meta-analysis of randomised controlled trials. Br. J. Ophthalmol..

[24] Guo H., Li X., Zhang X., Wang H., Li J. (2023). Comparing the effects of highly aspherical lenslets versus defocus incorporated multiple segment spectacle lenses on myopia control. Sci. Rep..

[25] Fan Y., Chu H., Peng Z., Zhou J., Ma J., Lu Y., Zhao C., Wang Y., Deng Q., Yu J. (2025). Real-world outcomes on myopia management efficacy of diverse segmented defocus optics (DSDO) and defocus incorporated multiple segments (DIMS) spectacle lenses in Chinese children: An initial 12-month prospective clinical study. J. Optom..

[26] Yan Y., Yang T., Tian Y., Du F., Yang Y., Chen R., Diao K., Wang J., Tao X., Yang J. (2026). Myopia control efficacy of annular microstructure defocus spectacle lenses: A 1-year multicenter study. BMC Ophthalmol..

[27] Yu Q., Zhou J.B. (2022). Scleral remodeling in myopia development. Int. J. Ophthalmol..

[28] Wang Z., Liu X., Zhang X., Sun B., Wang J., Hu X., Li Z., Lin W., Du B., Wei R. (2026). Association between first-year axial elongation and 10-year myopia progression in children wearing orthokeratology lenses: A ten-year longitudinal study. Front. Cell Dev. Biol..

[29] Zhang H., Lam C.S.Y., Tang W.C., Leung M., Qi H., Lee P.H., To C.H. (2022). Myopia Control Effect Is Influenced by Baseline Relative Peripheral Refraction in Children Wearing Defocus Incorporated Multiple Segments (DIMS) Spectacle Lenses. J. Clin. Med..

