# Supplementary material for: The Comparative Effectiveness of Four Myopia-Control Spectacle Lenses: A Real-World AIPW Cohort Study
Source: Healthcare (Basel). 2026 Sep 10;14(18):2958. doi: 10.3390/healthcare14182958 (PMC13606347; doi:10.3390/healthcare14182958)
Supplement: Supplementary file 1 [file healthcare-14-02958-s001.zip › healthcare-4451555-supplementary.pdf]

Table S1. Comparison of optical architecture among five spectacle lens groups.

Spectacle Lens Group	Optical Architecture	Presumed Design Mechanism	Peripheral Defocus Profile
SV(Single-Vision)	Standard monofocal optical lens	Corrects central refractive error only	No peripheral defocus signal (or hyperopic defocus)
DIMS (Defocus Incorporated Multiple Segments)	Central clear zone surrounded by a honeycomb area of micro-lenslets	Multiple discrete focal points in front of the retina	+3.50 D discrete peripheral myopic defocus
HAL (Highly Aspherical Lenslets)	Central clear zone surrounded by concentric rings of highly aspherical lenslets	Continuous volume of myopic defocus signal	Aspherical contiguous volume of peripheral defocus
DSDO (Diversified Segmental Defocus Optimization)	Central clear zone with multi-zone diversified segmental micro-structures	Optimized multi-segmental defocus distribution	Diversified segmental defocus distribution
HOAD (Higher-Order Aberration Defocus)	Central clear zone surrounded by dual-action micro-structures	Simultaneous peripheral myopic defocus + higher-order aberration (HOA) modulation	Integrated peripheral defocus with higher-order aberration modulation

Supplementary Table S2. Sensitivity analyses of pairwise comparisons across outcomes

Outcome	Comparison	Estimate (95% CI)	P_adj	Estimate (95% CI)	P_adj	Estimate (95% CI)	P_adj
		SA1: Trimming (0.5th–99.5th)		SA2: Trimming (2nd–98th)		SA3: PS Support (0.05–0.95)	
ΔAL	SV vs DIMS	0.082 (0.058, 0.107)	<0.001	0.082 (0.058, 0.107)	<0.001	0.081 (0.060, 0.102)	<0.001
	SV vs DSDO	0.052 (0.024, 0.080)	0.003	0.052 (0.024, 0.080)	0.003	0.048 (0.019, 0.078)	0.014
	SV vs HAL	0.109 (0.083, 0.134)	<0.001	0.109 (0.083, 0.134)	<0.001	0.107 (0.084, 0.131)	<0.001
	SV vs HOAD	0.150 (0.128, 0.172)	<0.001	0.150 (0.128, 0.172)	<0.001	0.148 (0.125, 0.172)	<0.001
	DIMS vs DSDO	-0.030 (-0.054, -0.006)	0.151	-0.030 (-0.054, -0.006)	0.151	-0.032 (-0.057, -0.007)	0.113
	DIMS vs HAL	0.026 (0.008, 0.044)	0.040	0.026 (0.008, 0.044)	0.040	0.026 (0.011, 0.042)	0.007
	DIMS vs HOAD	0.068 (0.055, 0.081)	<0.001	0.068 (0.055, 0.081)	<0.001	0.068 (0.054, 0.081)	<0.001
	DSDO vs HAL	0.057 (0.032, 0.082)	<0.001	0.057 (0.032, 0.082)	<0.001	0.059 (0.034, 0.084)	<0.001
	DSDO vs HOAD	0.098 (0.076, 0.120)	<0.001	0.098 (0.076, 0.120)	<0.001	0.100 (0.076, 0.124)	<0.001
	HAL vs HOAD	0.041 (0.026, 0.057)	<0.001	0.041 (0.026, 0.057)	<0.001	0.041 (0.026, 0.056)	<0.001
ΔSE	SV vs DIMS	-0.238 (-0.303, -0.173)	<0.001	-0.238 (-0.303, -0.173)	<0.001	-0.222 (-0.278, -0.166)	<0.001

	SV vs DSDO	-0.198 (-0.262, -0.133)	<0.001	-0.198 (-0.262, -0.133)	<0.001	-0.182 (-0.249, -0.114)	<0.001
	SV vs HAL	-0.260 (-0.327, -0.194)	<0.001	-0.260 (-0.327, -0.194)	<0.001	-0.245 (-0.303, -0.186)	<0.001
	SV vs HOAD	-0.368 (-0.428, -0.308)	<0.001	-0.368 (-0.428, -0.308)	<0.001	-0.352 (-0.407, -0.297)	<0.001
	DIMS vs DSDO	0.040 (-0.018, 0.098)	1	0.040 (-0.018, 0.098)	1	0.040 (-0.020, 0.100)	1
	DIMS vs HAL	-0.022 (-0.069, 0.024)	1	-0.022 (-0.069, 0.024)	1	-0.023 (-0.063, 0.017)	1
	DIMS vs HOAD	-0.130 (-0.167, -0.094)	<0.001	-0.130 (-0.167, -0.094)	<0.001	-0.130 (-0.166, -0.094)	<0.001
	DSDO vs HAL	-0.063 (-0.119, -0.006)	0.306	-0.063 (-0.119, -0.006)	0.306	-0.063 (-0.124, -0.002)	0.429
	DSDO vs HOAD	-0.170 (-0.220, -0.121)	<0.001	-0.170 (-0.220, -0.121)	<0.001	-0.170 (-0.224, -0.116)	<0.001
	HAL vs HOAD	-0.108 (-0.148, -0.068)	<0.001	-0.108 (-0.148, -0.068)	<0.001	-0.107 (-0.147, -0.068)	<0.001
Rapid AL Rate	SV vs DIMS	0.200 (0.134, 0.266)	<0.001	0.200 (0.134, 0.266)	<0.001	0.198 (0.135, 0.260)	<0.001
	SV vs DSDO	0.130 (0.044, 0.217)	0.032	0.130 (0.044, 0.217)	0.032	0.117 (0.036, 0.199)	0.048
	SV vs HAL	0.274 (0.204, 0.344)	<0.001	0.274 (0.204, 0.344)	<0.001	0.272 (0.205, 0.339)	<0.001
	SV vs HOAD	0.326 (0.268, 0.383)	<0.001	0.326 (0.268, 0.383)	<0.001	0.323 (0.258, 0.389)	<0.001
	DIMS vs DSDO	-0.070 (-0.144, 0.005)	0.670	-0.070 (-0.144, 0.005)	0.670	-0.081 (-0.156, -0.005)	0.365

	DIMS vs HAL	0.074 (0.025, 0.123)	0.031	0.074 (0.025, 0.123)	0.031	0.074 (0.022, 0.126)	0.055
	DIMS vs HOAD	0.126 (0.082, 0.170)	<0.001	0.126 (0.082, 0.170)	<0.001	0.126 (0.084, 0.167)	<0.001
	DSDO vs HAL	0.144 (0.067, 0.220)	0.002	0.144 (0.067, 0.220)	0.002	0.155 (0.078, 0.232)	<0.001
	DSDO vs HOAD	0.195 (0.127, 0.264)	<0.001	0.195 (0.127, 0.264)	<0.001	0.206 (0.140, 0.272)	<0.001
	HAL vs HOAD	0.052 (0.003, 0.100)	0.364	0.052 (0.003, 0.100)	0.364	0.052 (0.005, 0.098)	0.297
Rapid SE Rate	SV vs DIMS	0.216 (0.150, 0.283)	<0.001	0.216 (0.150, 0.283)	<0.001	0.212 (0.149, 0.274)	<0.001
	SV vs DSDO	0.196 (0.114, 0.278)	<0.001	0.196 (0.114, 0.278)	<0.001	0.189 (0.112, 0.267)	<0.001
	SV vs HAL	0.262 (0.194, 0.330)	<0.001	0.262 (0.194, 0.330)	<0.001	0.258 (0.191, 0.324)	<0.001
	SV vs HOAD	0.322 (0.262, 0.381)	<0.001	0.322 (0.262, 0.381)	<0.001	0.317 (0.258, 0.377)	<0.001
	DIMS vs DSDO	-0.020 (-0.092, 0.052)	1	-0.020 (-0.092, 0.052)	1	-0.022 (-0.096, 0.051)	1
	DIMS vs HAL	0.046 (-0.007, 0.098)	0.868	0.046 (-0.007, 0.098)	0.868	0.046 (-0.006, 0.098)	0.799
	DIMS vs HOAD	0.106 (0.064, 0.147)	<0.001	0.106 (0.064, 0.147)	<0.001	0.106 (0.067, 0.144)	<0.001
	DSDO vs HAL	0.066 (-0.009, 0.140)	0.829	0.066 (-0.009, 0.140)	0.829	0.068 (-0.006, 0.143)	0.731
	DSDO vs HOAD	0.125 (0.062, 0.189)	0.001	0.125 (0.062, 0.189)	0.001	0.128 (0.060, 0.195)	0.002

HAL vs HOAD	0.060 (0.010, 0.110)	0.194	0.060 (0.010, 0.110)	0.194	0.059 (0.011, 0.108)	0.154

SA1: Inverse probability of treatment weighting with weight trimming at the 0.5th and 99.5th percentiles.

SA2: Inverse probability of treatment weighting with weight trimming at the 2nd and 98th percentiles.

SA3: Restriction to the common propensity-score support region (0.05–0.95).

Table S3. AIPW Diagnostic Summary: Covariate Balance, Weight Distribution, Effective Sample Size, and Positivity Assessment

Panel A. Covariate Balance Diagnostics by Treatment Comparison

Comparison	Max |SMD| (Pre)	Max |SMD| (Post)	Covariate (Max Post)	Post-SMD Value	Balanced (Post <0.10)	Covariates >0.10 (Post)
DIMS vs. SV	0.272	0.107	Year	+0.1069	No	1
DSDO vs. SV	0.384	0.084	Year	+0.0837	Yes	0
HAL vs. SV	0.281	0.140	Year	+0.1398	No	1
HOAD vs. SV	0.263	0.115	Year	+0.1155	No	1
DSDO vs. DIMS	0.594	0.109	LT	+0.1087	No	1
HAL vs. DIMS	0.115	0.033	Year	+0.0330	Yes	0
HOAD vs. DIMS	0.107	0.018	se0	-0.0176	Yes	0
HAL vs. DSDO	0.622	0.117	LT	-0.1174	No	1
HOAD vs. DSDO	0.558	0.099	LT	-0.0989	Yes	0
HOAD vs. HAL	0.082	0.030	se0	-0.0296	Yes	0

Panel B. Weight Distribution and Effective Sample Size (ESS) by Treatment Group

Group	N	Weight Median (Q1, Q3)	Weight Mean ± SD	Weight Range	ESS (Unadjust	ESS (Adjusted) (Retention %)

				(Min–Max)	ed)	
SV	338	8.09 (5.96, 10.14)	8.33 ± 2.91	3.69–17.04	338	297.3 (88.0%)
DIMS	800	3.38 (3.06, 3.73)	3.44 ± 0.51	2.60–5.16	800	781.5 (97.7%)
DSDO	238	11.53 (8.68, 14.43)	11.41 ± 4.48	1.50–20.84	238	205.6 (86.4%)
HAL	476	5.62 (5.07, 6.29)	5.76 ± 0.95	3.91–8.49	476	462.4 (97.1%)
HOAD	912	3.00 (2.85, 3.16)	3.02 ± 0.25	2.53–3.84	912	905.0 (99.2%)

Panel C. Trimming and Positivity / Common Support Assessment

Metric	Value
Total Sample (N)	2764
Trimmed Count (PS <0.05 or >0.95)	7
Percentage Trimmed (%)	0.25%
Winsorization Lower/Upper	1% / 99%

Note. AIPW = augmented inverse probability weighting; SMD = standardized mean difference; ESS = effective sample size; PS = propensity score. Balance threshold: |SMD| < 0.10. Positivity assessed by trimming observations with PS < 0.05 or > 0.95, followed by Winsorization at 1st and 99th percentiles. Pairwise covariate-specific pre- and post-weighting SMDs for all 10 comparisons and 8 covariates are provided in Table S2.

Table S4. Pairwise Covariate-Specific Standardized Mean Differences (SMD), Pre- and Post-AIPW Weighting

Comparison	Covariate	SMD (Unweighted)	SMD (Weighted)	|SMD| Reduction (%)
DIMS vs. SV	Year	-0.2716	+0.1069	60.7%
DIMS vs. SV	Sex	-0.0285	-0.0150	47.4%
DIMS vs. SV	AL0	-0.0440	-0.0132	70.0%
DIMS vs. SV	se0	+0.2098	+0.0484	76.9%
DIMS vs. SV	K	-0.1565	-0.0337	78.5%
DIMS vs. SV	ACD	-0.0031	+0.0336	-976.2%
DIMS vs. SV	CCT	+0.0429	+0.0179	58.4%
DIMS vs. SV	LT	+0.0382	-0.0687	-79.9%
DSDO vs. SV	Year	-0.3331	+0.0837	74.9%
DSDO vs. SV	Sex	+0.0330	-0.0147	55.3%
DSDO vs. SV	AL0	+0.2284	-0.0056	97.5%
DSDO vs. SV	se0	-0.3843	-0.0185	95.2%
DSDO vs. SV	K	-0.0221	-0.0075	65.9%
DSDO vs. SV	ACD	-0.0774	+0.0294	62.1%
DSDO vs. SV	CCT	+0.0039	+0.0174	-344.5%
DSDO vs. SV	LT	+0.1006	+0.0400	60.3%
HAL vs. SV	Year	-0.2815	+0.1398	50.3%
HAL vs. SV	Sex	-0.0343	-0.0189	44.9%
HAL vs. SV	AL0	-0.1338	-0.0070	94.8%
HAL vs. SV	se0	+0.2375	+0.0604	74.6%
HAL vs. SV	K	-0.1310	-0.0459	65.0%
HAL vs. SV	ACD	-0.1186	+0.0216	81.8%
HAL vs. SV	CCT	+0.1301	+0.0470	63.9%
HAL vs. SV	LT	-0.0018	-0.0774	-4197.4%
HOAD vs. SV	Year	-0.2634	+0.1155	56.2%
HOAD vs. SV	Sex	-0.0121	-0.0144	-18.6%
HOAD vs. SV	AL0	-0.1039	+0.0014	98.7%
HOAD vs. SV	se0	+0.1742	+0.0308	82.3%
HOAD vs. SV	K	-0.0491	-0.0331	32.7%

Comparison	Covariate	SMD (Unweighted)	SMD (Weighted)	|SMD| Reduction (%)
HOAD vs. SV	ACD	-0.0372	+0.0370	0.6%
HOAD vs. SV	CCT	+0.0852	+0.0219	74.3%
HOAD vs. SV	LT	+0.0790	-0.0589	25.4%
DSDO vs. DIMS	Year	-0.0615	-0.0231	62.4%
DSDO vs. DIMS	Sex	+0.0614	+0.0002	99.6%
DSDO vs. DIMS	AL0	+0.2724	+0.0076	97.2%
DSDO vs. DIMS	se0	-0.5940	-0.0669	88.7%
DSDO vs. DIMS	K	+0.1344	+0.0261	80.6%
DSDO vs. DIMS	ACD	-0.0743	-0.0043	94.2%
DSDO vs. DIMS	CCT	-0.0390	-0.0005	98.8%
DSDO vs. DIMS	LT	+0.0624	+0.1087	-74.2%
HAL vs. DIMS	Year	-0.0098	+0.0330	-234.8%
HAL vs. DIMS	Sex	-0.0058	-0.0039	32.4%
HAL vs. DIMS	AL0	-0.0898	+0.0063	93.0%
HAL vs. DIMS	se0	+0.0277	+0.0120	56.8%
HAL vs. DIMS	K	+0.0255	-0.0122	52.0%
HAL vs. DIMS	ACD	-0.1155	-0.0120	89.6%
HAL vs. DIMS	CCT	+0.0872	+0.0291	66.6%
HAL vs. DIMS	LT	-0.0400	-0.0087	78.2%
HOAD vs. DIMS	Year	+0.0082	+0.0086	-4.9%
HOAD vs. DIMS	Sex	+0.0163	+0.0006	96.6%
HOAD vs. DIMS	AL0	-0.0599	+0.0146	75.7%
HOAD vs. DIMS	se0	-0.0356	-0.0176	50.4%
HOAD vs. DIMS	K	+0.1074	+0.0006	99.5%
HOAD vs. DIMS	ACD	-0.0341	+0.0033	90.2%
HOAD vs. DIMS	CCT	+0.0423	+0.0040	90.5%
HOAD vs. DIMS	LT	+0.0408	+0.0098	75.9%
HAL vs. DSDO	Year	+0.0516	+0.0561	-8.7%
HAL vs. DSDO	Sex	-0.0672	-0.0041	93.8%
HAL vs. DSDO	AL0	-0.3622	-0.0013	99.6%

Comparison	Covariate	SMD (Unweighted)	SMD (Weighted)	|SMD| Reduction (%)
HAL vs. DSDO	se0	+0.6218	+0.0788	87.3%
HAL vs. DSDO	K	-0.1089	-0.0384	64.8%
HAL vs. DSDO	ACD	-0.0412	-0.0077	81.2%
HAL vs. DSDO	CCT	+0.1262	+0.0296	76.6%
HAL vs. DSDO	LT	-0.1024	-0.1174	-14.7%
HOAD vs. DSDO	Year	+0.0697	+0.0317	54.4%
HOAD vs. DSDO	Sex	-0.0451	+0.0003	99.3%
HOAD vs. DSDO	AL0	-0.3323	+0.0070	97.9%
HOAD vs. DSDO	se0	+0.5585	+0.0493	91.2%
HOAD vs. DSDO	K	-0.0270	-0.0255	5.5%
HOAD vs. DSDO	ACD	+0.0402	+0.0076	81.1%
HOAD vs. DSDO	CCT	+0.0813	+0.0045	94.5%
HOAD vs. DSDO	LT	-0.0216	-0.0989	-358.0%
HOAD vs. HAL	Year	+0.0180	-0.0244	-35.0%
HOAD vs. HAL	Sex	+0.0221	+0.0045	79.7%
HOAD vs. HAL	AL0	+0.0298	+0.0083	72.1%
HOAD vs. HAL	se0	-0.0633	-0.0296	53.2%
HOAD vs. HAL	K	+0.0819	+0.0128	84.3%
HOAD vs. HAL	ACD	+0.0814	+0.0153	81.1%
HOAD vs. HAL	CCT	-0.0449	-0.0251	44.1%
HOAD vs. HAL	LT	+0.0808	+0.0185	77.1%

Note. Pre-weighting SMD reflects raw group differences; post-weighting SMD reflects balance after AIPW weighting. Reduction % = (1 − |post-SMD| / |pre-SMD|) × 100. Covariates: Year = age at baseline; Sex = biological sex; AL0 = baseline axial length; se0 = baseline spherical equivalent; K = corneal curvature; ACD = anterior chamber depth; CCT = central corneal thickness; LT = lens thickness.

Table S5A. Subgroup analysis of the 1-year ATEs of the four myopia-control designs versus SV lenses on ΔAL.

Subgroup Type	Subgroup Level	Comparison	Total N	SV N	Intervention N	ATE (95% CI)	P value	Adjusted P

Age	≤6 years	DIMS	80	18	20	-0.0069 (-5.2096, 5.1959)	0.998	1.000
		DSDO	80	18	12	0.7144 (-53.8649, 55.2937)	0.980	1.000
		HAL	80	18	8	0.3543 (-252.0243, 252.7329)	0.998	1.000
		HOAD	80	18	22	0.1859 (-2.5619, 2.9336)	0.895	1.000
	7-12 years	DIMS	2018	192	600	-0.0988 (-0.1287, -0.0689)	<0.001	**<0.001**
		DSDO	2018	192	178	-0.0749 (-0.1130, -0.0368)	0.001	**0.001**
		HAL	2018	192	366	-0.1364 (-0.1695, -0.1033)	<0.001	**<0.001**
		HOAD	2018	192	682	-0.1845 (-0.2148, -0.1543)	<0.001	**<0.001**
	>12 years	DIMS	666	128	180	-0.0504 (-0.0802, -0.0205)	0.001	**0.012**
		DSDO	666	128	48	-0.0555 (-0.1061, -0.0049)	0.032	0.380
		HAL	666	128	102	-0.0878 (-0.1196, -0.0561)	<0.001	**<0.001**
		HOAD	666	128	208	-0.0904 (-0.1194, -0.0614)	<0.001	**<0.001**
Baseline SE	Mild Myopia	DIMS	2051	237	611	-0.0951 (-0.1213, -0.0690)	<0.001	**<0.001**
		DSDO	2051	237	139	-0.0671 (-0.1008, -0.0335)	0.001	**0.001**
		HAL	2051	237	360	-0.1343 (-0.1614, -0.1072)	<0.001	**<0.001**
		HOAD	2051	237	704	-0.1753 (-0.2013, -0.1494)	<0.001	**<0.001**
	Moderate Myopia	DIMS	581	79	170	-0.0011 (-0.0594, 0.0573)	0.971	1.000
		DSDO	581	79	68	0.0336 (-0.0393, 0.1064)	0.367	1.000
		HAL	581	79	106	-0.0189 (-0.0817, 0.0439)	0.555	1.000
		HOAD	581	79	158	-0.0780 (-0.1381, -0.0180)	0.011	0.130
	Severe Myopia	DIMS	132	22	19	0.0093 (-1.3389, 1.3575)	0.989	1.000
		DSDO	132	22	31	-0.1496 (-0.7807, 0.4815)	0.642	1.000
		HAL	132	22	10	0.0096 (-42.6532, 42.6724)	1.000	1.000
		HOAD	132	22	50	0.0614 (-0.5767, 0.6995)	0.850	1.000
Baseline AL	Normal/Short AL	DIMS	852	110	238	-0.0844 (-0.1254, -0.0434)	<0.001	**<0.001**
		DSDO	852	110	52	-0.0509 (-0.1173, 0.0154)	0.133	1.000
		HAL	852	110	140	-0.1153 (-0.1597, -0.0709)	<0.001	**<0.001**
		HOAD	852	110	312	-0.1848 (-0.2266, -0.1429)	<0.001	**<0.001**
	Intermediate AL	DIMS	1726	201	514	-0.0872 (-0.1166, -0.0578)	<0.001	**<0.001**
		DSDO	1726	201	159	-0.0499 (-0.0863, -0.0136)	0.007	0.085
		HAL	1726	201	310	-0.1133 (-0.1446, -0.0821)	<0.001	**<0.001**
		HOAD	1726	201	542	-0.1403 (-0.1719, -0.1088)	<0.001	**<0.001**

		DIMS	186	27	48	-0.0682 (-0.3718, 0.2355)	0.660	1.000
	Pathological AL	DSDO	186	27	27	-0.0755 (-0.4670, 0.3160)	0.705	1.000
		HAL	186	27	26	-0.0921 (-0.4287, 0.2444)	0.592	1.000
		HOAD	186	27	58	-0.0725 (-0.3860, 0.2410)	0.650	1.000

Note. All intervention groups were compared with single-vision (SV) lenses. Negative ATE indicates less axial elongation in the intervention group compared with SV.

Table S5B. Subgroup analysis of the 1-year ATEs of the four myopia-control designs versus SV lenses on ΔSE.

**Subgroup Type**	**Subgroup Level**	**Comparison**	**Total N**	**SV N**	**Intervention N**	**ATE (95% CI)**	**P value**	**Adjusted P**
Age	≤6 years	DIMS	80	18	20	-0.1776 (-16.2257, 15.8706)	0.983	1.000
		DSDO	80	18	12	-2.5999 (-302.5980, 297.3981)	0.986	1.000
		HAL	80	18	8	-0.4465 (-639.4790, 638.5864)	0.999	1.000
		HOAD	80	18	22	-0.8979 (-12.3199, 10.5241)	0.878	1.000
	7-12 years	DIMS	2018	192	600	0.2495 (0.1739, 0.3251)	<0.001	**<0.001**
		DSDO	2018	192	178	0.2483 (0.1553, 0.3412)	<0.001	**<0.001**
		HAL	2018	192	366	0.3244 (0.2429, 0.4060)	<0.001	**<0.001**
		HOAD	2018	192	682	0.4302 (0.3546, 0.5058)	<0.001	**<0.001**
	>12 years	DIMS	666	128	180	0.2151 (0.1157, 0.3144)	<0.001	**<0.001**
		DSDO	666	128	48	0.2307 (0.0916, 0.3699)	0.001	**0.014**
		HAL	666	128	102	0.2138 (0.1081, 0.3194)	<0.001	**<0.001**
		HOAD	666	128	208	0.3111 (0.2172, 0.4050)	<0.001	**<0.001**
Baseline SE	Mild Myopia	DIMS	2051	237	611	0.2555 (0.1851, 0.3260)	<0.001	**<0.001**
		DSDO	2051	237	139	0.2371 (0.1438, 0.3304)	<0.001	**<0.001**
		HAL	2051	237	360	0.3138 (0.2396, 0.3881)	<0.001	**<0.001**
		HOAD	2051	237	704	0.4124 (0.3437, 0.4811)	<0.001	**<0.001**
	Moderate Myopia	DIMS	581	79	170	0.0755 (-0.0972, 0.2481)	0.857	0.392
		DSDO	581	79	68	0.0499 (-0.1613, 0.2612)	0.643	1.000
		HAL	581	79	106	0.0386 (-0.1427, 0.2199)	0.677	1.000
		HOAD	581	79	158	0.2185 (0.0411, 0.3959)	0.016	0.189
	Severe	DIMS	132	22	19	0.1819 (-2.2412, 2.6050)	0.883	1.000

	Myopia	DSDO	132	22	31	0.2967 (-1.1594, 1.7527)	0.690	1.000
		HAL	132	22	10	-0.7077 (-43.8476, 42.4322)	0.974	1.000
		HOAD	132	22	50	0.0537 (-1.4442, 1.5516)	0.944	1.000
Baseline AL	Normal/Short AL	DIMS	852	110	238	0.2042 (0.0952, 0.3132)	<0.001	**0.003**
		DSDO	852	110	52	0.1922 (0.0270, 0.3575)	0.023	0.271
		HAL	852	110	140	0.3060 (0.1904, 0.4216)	<0.001	**<0.001**
		HOAD	852	110	312	0.4554 (0.3409, 0.5700)	<0.001	**<0.001**
	Intermediate AL	DIMS	1726	201	514	0.2986 (0.1926, 0.4046)	<0.001	**<0.001**
		DSDO	1726	201	159	0.2369 (0.1231, 0.3507)	<0.001	**<0.001**
		HAL	1726	201	310	0.2940 (0.1832, 0.4047)	<0.001	**<0.001**
		HOAD	1726	201	542	0.3803 (0.2706, 0.4899)	<0.001	**<0.001**
	Pathological AL	DIMS	186	27	48	0.2582 (-1.6552, 2.1716)	0.265	0.791
		DSDO	186	27	27	0.2472 (-1.7354, 2.2299)	0.244	0.807
		HAL	186	27	26	0.1757 (-1.7736, 2.1250)	0.177	0.860
		HOAD	186	27	58	0.3061 (-1.6175, 2.2297)	0.312	0.755

Note. All intervention groups were compared with single-vision (SV) lenses. Positive ATE indicates less myopic shift (slower SE progression) in the intervention group compared with SV.

Table S6. Tests for interaction between treatment assignment and subgroup variables on 12-month axial length and spherical equivalent changes.

Outcome	Subgroup Variable	χ^2	df	P for Interaction	Significant
ΔAL (mm)	Sex	1.664	4	0.797	No
	Age	42.830	8	**<0.001**	Yes
	Baseline SE	38.456	8	**<0.001**	Yes
	Baseline AL	18.200	8	**0.020**	Yes
ΔSE (D)	Sex	5.309	4	0.257	No
	Age	28.212	8	**<0.001**	Yes
	Baseline SE	23.703	8	**0.003**	Yes
	Baseline AL	18.605	8	**0.017**	Yes

Note. χ^2 = likelihood-ratio chi-square statistic from AIPW-weighted interaction models.
